# Neuroenhancement of High-Level Cognition: Evidence for Homeostatic Constraints of Non-invasive Brain Stimulation

**DOI:** 10.1007/s41465-019-00126-7

**Published:** 2019-02-21

**Authors:** Beatrix Krause, Martin Dresler, Chung Yen Looi, Amar Sarkar, Roi Cohen Kadosh

**Affiliations:** 1 Department of Experimental Psychology, University of Oxford, Oxford, UK; 2 Late-Life Mood, Stress, and Wellness Research Program, Semel Insitute for Neuroscience and Human Behavior, Geffen School of Medicine at UCLA, 760 Westwood Plaza, Los Angeles, CA 90095, USA; 3 Donders Institute for Brain, Cognition and Behaviour, Radboud University, Nijmegen, The Netherlands

**Keywords:** Brain stimulation, Cognition, Calculation, Prodigy

## Abstract

Neuroenhancement aims to improve cognitive performance in typically and atypically functioning populations. However, it is currently debated whether it is also effective in exceptionally high-functioning individuals. Present theories suggest that homeostatic set points for learning and cortical plasticity limit the beneficial effects of neuroenhancement. To examine this possibility, we used transcranial random noise stimulation (tRNS) to non-invasively stimulate bilateral dorsolateral prefrontal cortices (DLPFC) of the world champion in mental calculation, G.M. TRNS did not change G.M.’s calculation performance compared to sham stimulation on an exceptionally complex arithmetic task. However, a sample of mathematicians who were not calculation prodigies (*N* = 6) showed reduced accuracy on a complex multiplication task in response to tRNS, relative to sham. Our findings suggest that there may be an upper limit for cognitive enhancement and that further attempts to enhance performance using tRNS (at least with the current parameters) may impair optimal functioning. The discussion of potential negative effects of brain stimulation for cognitive enhancement is critical, as it may lead to unintended impairments in different subgroups of the population.

How far can we improve human mental performance? Teaching and training often succeed in improving performance, presumably via changes in brain functioning. More recently, however, it has become possible to influence brain function more directly. Methods such as non-invasive brain stimulation (NIBS) can lead to long-term cognitive improvements, and enhance cognitive performance in healthy individuals (Dayan et al. 2013; Krause and Cohen Kadosh 2013; Santarnecchi et al. 2015). NIBS is, nowadays, mostly aimed to improve abilities in individuals with neuropsychological deficits, including for instance, cognitive and motor impairments after stroke or atypical development (Costanzo et al. 2016; Fiori et al. 2011; Fridriksson et al. 2011; Holland and Crinion 2012; Looi et al. 2017; Reis and Fritsch 2011; You et al. 2011). Low-performing individuals often appear to benefit most from such neuromodulation (Looi et al. 2016; E. Santarnecchi et al. 2016; Sarkar et al. 2014; Tseng et al. 2012). Since the introduction of NIBS in the research and clinical environment, we also have to consider how its use will affect different individuals, including high-functioning ones. We have previously suggested that the effects of transcranial electrical stimulation (tES), a form of NIBS, depend on individual differences in a range of variables, including age, sex, brain state, and regional neurotransmitter levels (Krause and Cohen Kadosh 2014). This means that besides possible improvements, there is the potential for individuals with extraordinary cognitive ability to experience either no gain from the stimulation, or even experience impairment.

For both the recipient of the stimulation and the public, it is important that we understand the consequences of NIBS in high-performing individuals. The media nowadays promote “brain-boosting” methods, especially nonpharmacological cognitive enhancement (Dresler et al. 2013). The hope is for NIBS to push the boundaries of the human brain and cognition further than possible at present. The consequences of such actions are unpredictable at this point, and there is a risk of arousing unrealistic expectations. Furthermore, if NIBS impairs the abilities of some high-functioning individuals, this technology may unintentionally affect their future educational or occupational functioning. Low-cost self-stimulation kits for home use are already on the market (Maslen et al. 2013, 2014); therefore, there is a particular need to explore the limits of the technique in order to avoid potential abuse and protect the unskilled user from unwanted and possibly irreversible damage (Cohen Kadosh et al. 2012). It is also important to educate the population about both risks and benefits of this method.

Present theories suggest that the effect of NIBS on cortical plasticity is limited by homeostatic set point mechanisms, such that an individual’s capacity cannot be exceeded due to counter-regulation by the brain’s natural balance of plasticity (Siebner et al. 2004). This means that there is a hypothetical optimal level of change, but if a brain region is pushed toward excessive levels of excitation, counter-mechanisms will be initiated to avoid further changes. Consequently, progressing beyond a given capacity may not be feasible according to the homeostatic set point theory. In line with this theory, we have recently suggested that individual differences in the capacity for plasticity determine the outcome of the stimulation and that brains with high levels of capacity and functioning are more likely to show either no effect or even impairments resulting from the stimulation (Krause and Cohen Kadosh 2014; Krause et al. 2013). It is possible that an individual with close-to-boundary capacity experiences no further improvements from plasticity-enhancing methods.

Individuals with prodigious abilities have fascinated psychologists and the public for over a century, but little is known about the underlying mechanisms of exceptional mental abilities. One of the most sophisticated of human abilities is mental calculation (Butterworth 1999). Calculation prodigies are characterized by extraordinary memory recall or arithmetic processing speed (“lightning calculators”) and their high level of accuracy in solving highly complex mathematical problems (Snyder and Mitchell 1999). Both structural and functional differences have been found in brain regions supporting working memory and episodic memory capacity of such individuals compared to normal calculators (Fehr et al. 2010). In particular, the dorsolateral prefrontal cortex (DLPFC) is one of the core regions showed elevated brain activity in a calculation prodigy (Pesenti et al. 2001). The question arises as to whether we can enhance brain functioning in individuals with extraordinary abilities, which could not otherwise be reached by practice or strategy use. We examined this possibility by applying transcranial random noise stimulation (tRNS), a form of tES to enhance mental calculation skills in a single case of the world’s leading mental calculation prodigy (G.M.). G.M. has been practicing mental calculation since the age of three and has continuously demonstrated his abilities by winning successive world records and world championships. We have previously found that the excitatory/inhibitory balance between neurotransmitters involved in learning and memory was altered in G.M.’s DLPFC compared to that of nonprodigious mathematically skilled individuals (Krause et al. 2018).

We hypothesized that G.M.’s calculation abilities would further improve with tRNS. This method excites two brain areas simultaneously (i.e., bilateral DLPFC), with a high perceptual cutaneous threshold (less noticeable to the recipient) to ascertain proper participant-blinding (Ambrus et al. 2010). A proposed mechanism of random noise is stochastic resonance (Fertonani et al. 2011; van der Groen and Wenderoth 2016). A subthreshold stimulus (in this case, weak neuronal activity) can reach the threshold when noise is added (Moss et al. 2004). Such an effect results in an increase in synchronous neuronal firing, which may affect cognition. For example, tRNS over the bilateral DLPFC improved arithmetic performance in typical adult participants and the effect was still stable at a 6-month follow-up investigation (Snowball et al. 2013). However, an alternative prediction based on the stochastic resonance framework is that when noise is applied to a supratreshold signal, the effect can be absent or even detrimental (van der Groen and Wenderoth 2016).

In another experiment (experiment 2), we invited a small sample (*N* = 6) of postgraduate students in the field of mathematics, statistics, and engineering, who performed a complex multiplication task while receiving tRNS. The motivation of this experiment was to examine the effect of NIBS on above-average, even if not prodigious, individuals. Since this sample of participants would be unable to perform G.M.’s complex calculation task, the task was designed to compare whether these highly functioning individuals would show the same direction of effects as G.M.

## Experiment 1: Calculation Expert

### Methods

#### Participant

G.M. is a male German, 46-year-old, high-functioning, healthy calculation expert with no history of neurological or psychiatric conditions. He has been engaging in competitive mental calculation events for more than 25 years and is a member of Mensa, The High IQ Society. G.M.’s exceptional skills have repeatedly been demonstrated in international mental calculation competitions such as the Mental Calculation World Cup or the Mind Sports Olympiad (MSO). G.M. is an 11-time winner of the MSO mental calculation gold medal, and he holds several world records in mental calculation. He holds two university doctorate degrees in humanities (education and psychology), a master’s degree in computer science, and is also highly skilled in calendrical calculation. His standardized mathematical abilities lie above the 99.8th percentile (top composite standard score = 143, WIAT®-II^UK^ (see, Wechsler 2005), and also in measurements of fluid reasoning and mental speed, he scored above the 99.9th percentile, resulting in an extrapolated IQ estimation of above 160 (see, Oswald and Roth 1987; Weiß and Weiß 2006). G.M. provided written informed consent prior to the beginning of the study. The study was approved by the Berkshire Research Ethics Committee.

#### Standardized Batteries

We assessed G.M.’s basic mathematical abilities prior to the stimulation experiment. The Wechsler Individual Achievement Test (WIAT®-II^UK^) assesses standardized measures for basic mathematical abilities (Wechsler 2005). The WIAT-II comprises two subscales. The “numerical operations” subscale provides increasingly difficult arithmetic problems, while the “mathematical reasoning” subscale tests the logical application of mathematical rules to increasingly difficult verbal and visual mathematical problems. We computed the age-appropriate standard scores according to the Wechsler scoring tables. A composite score can be computed from the combined standard scores of the two subtests.

Fluid reasoning was assessed using the Culture Fair Intelligence Test, which eliminates potential cultural influences on intelligence measurements (CFT-20R; Weiß and Weiß 2006). As G.M. exceeded the standardized scores of IQ measures, his IQ was estimated to be above 160. Furthermore, G.M.’s mental speed was measured using a trail-making test (ZVT; Oswald and Roth 1987). Again, G.M. exceeded the standardized scores, with an extrapolated IQ estimate above 160.

#### Stimulation Task

**Fig. 1:**
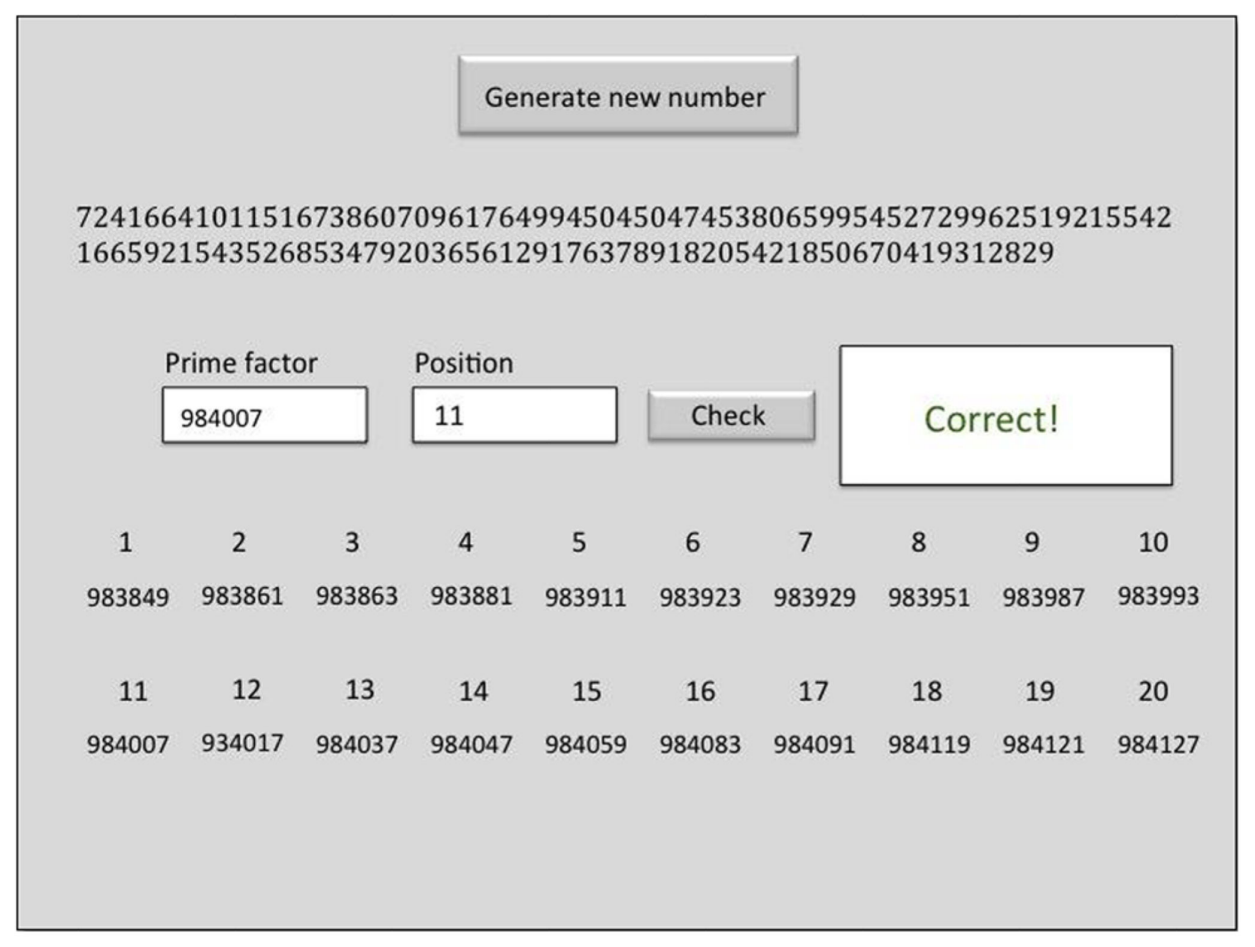
G.M.’s calculation task: a 120-digit number (top) was generated upon button click. This number was the product of 20 successively multiplied six-digit prime numbers (positions 1–20 displayed here). One of these had to be identified and entered as quickly as possible. In more complex trials, the exact position of the prime factor in the succession of multiplications had to be additionally identified. The task provided feedback on the correctness of the response

The task consisted of highly complex calculations that could not be performed by individuals with normal, or even advanced calculation skills. The problems presented on a computer screen involved either a 100- or a 120-digit number that had to be broken down into 20 different 6-digit prime number factors that, multiplied by each other in ascending order, made up the presented number (i.e., prime factor 1 × prime factor 2 × […]× prime factor 20 equaled this large number). G.M. had to either successfully identify one of these twenty prime numbers, or additionally provide the factor position within the multiplication sequence (1–20; Fig. 1). Throughout the task, G.M. had lists of all prime numbers up to 1,000,000 in front of him in order to confirm that his final answer comprised of an existing prime number. It is important to note that some individuals have the prodigious ability to recognize or produce large prime numbers while being incapable of performing mental arithmetic (Welling 1994). G.M.’s expertise is specifically in rapid mental calculation and he has not memorized all 78,499 prime numbers between 1 and 1 million. Note that in such a rare single-case study, it is extremely challenging or impossible to have a control group that would be able to solve the same task problems. Therefore, the best practice in this case is to use the participant as his own control (Cohen Kadosh et al. 2008; Sapir et al. 1999).

#### Procedure

On two consecutive days, G.M. underwent tRNS (0.1–500 Hz frequency range) in eight double-blind sessions using a wireless StarStim Neuroelectrics® stimulator (Barcelona, Spain). TRNS and sham stimulation were applied in a randomized order. The current (1 mA) was delivered by two circular electrodes of 25 cm^2^ each to F3 and F4 electrode positions, according to the international 10–20 system for EEG recording. TRNS was applied during the administration of the calculation task for 20 min per session with 15 s ramp up and ramp down. Sham stimulation consisted of 30 s of stimulation with 15 s ramp up and ramp down to mimic the skin sensations experienced during real stimulation. Due to a software crash, the first session (tRNS) terminated after a few minutes and the data had to be discarded. After a short break, the session was resumed with the same stimulation parameters. By the beginning of the 8th session, G.M. reported severe concentration problems, such that the session was also terminated. Accordingly, six full sessions of data over the course of two days were available for analysis. G.M. reported no skin sensations under the electrodes during any of the sessions, supporting the idea of higher cutaneous perception threshold compared to other forms of electrical stimulation (Ambrus et al. 2010). G.M. was also unable to guess the stimulation conditions in all cases. The task order was tRNS, sham, tRNS, sham, sham, tRNS.

### Results

**Fig. 2:**
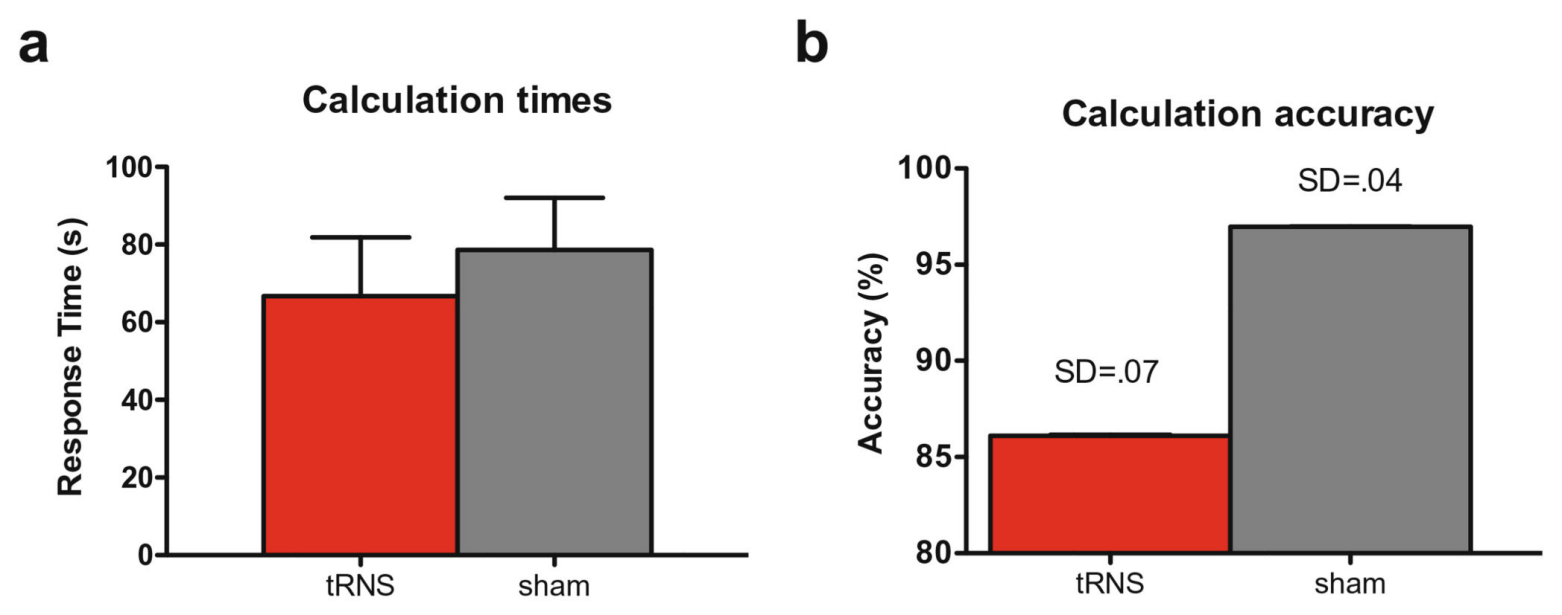
G.M.’s calculation performance during tRNS stimulation and sham control (mean and standard deviation). **a** There was no effect of stimulation on (**a**) calculation times in seconds or B) accuracy (in percent). Note that the standard deviation in (**b**) does not show due to the scaling

During each session, G.M. performed complex calculations at four different levels of difficulty. The order of levels of difficulty was randomized across sessions. Trials that were answered incorrectly were excluded from the analysis (8.6% with no significant differences between the number of trials under real (*N* = 4) and sham (*N* = 5); Fisher’s exact test, *p* > .2). Response times from problem presentation until logging in of G.M.’s answers were recorded by two experimenters. One trial was excluded from the analysis due to an accidental difference of 50 s between the observers and was attributed to an experimental error. Scores were computed for each trial as the average times from the two observers. The correlation between the observers recorded time was .99 (Spearman’s rho, *t*(53) = 95.02; *p* < .001). Outliers were removed if calculation times exceeded more than 2.5 standard deviations of the mean. This strategy ascertained that the recorded response times represented actual calculation times instead of distraction of the participant. Three trials were removed in the sham condition and two in tRNS. For inferential statistics, each trial was considered a case, resulting in 76 trials in total. TRNS and sham calculation times were not correlated (*r_ρ_* = −.24; *p* = .15). Paired-samples or repeated measures tests were not justified due to the unequal numbers of trials per condition (sham *N* = 41; tRNS *N* = 35). A one-way analysis of variance (ANOVA) demonstrated that sham and tRNS did not differ in their calculation times (*F*(1,74) = .35; *p* = .56; Fig. 2a). The result was the same using the non-parametric related-samples Wilcoxon signed-rank test (*W*(35) = 5344.5; *Z* = 25; *p* = .8). A Wilcoxon signed-rank test on accuracy on the three sessions per condition revealed no significant difference either (*W* < .001; *Z* = − 1.63; *p* = .1; Fig. 2b).

### Intermediate Discussion

Using tRNS to increase cortical excitability in the bilateral DLPFC of G.M. did not affect calculation times. Accuracy decreased by almost 11% under tRNS compared to sham, but the difference was not statistically significant. It is also important to note that the reduction in accuracy was accompanied by an increase in calculation times. Therefore, a speed-accuracy trade-off may have developed over the course of the stimulation period. Unfortunately, G.M., who resides in a different country, was not available for further sessions on other days, which could have enhanced control over experimental testing conditions. This was a single-case study with a unique participant, which introduced challenges in data acquisition, statistical analysis and subsequent interpretation of the results. Therefore, it is difficult to draw firm conclusions on these observations. G.M. cannot be compared to normal, or even highly proficient calculators, based on the complexity of the task at hand. In order to further investigate how tRNS affects individuals with high arithmetic abilities, we tested an additional sample of six healthy postgraduate students with high standardized mathematics scores.

## Experiment 2: Mathematicians

### Methods

#### Participants

Six healthy postgraduate students in the field of mathematics and statistics from the University of Oxford were recruited to represent a mathematically highly proficient sample (details see Table 1). Written informed consent was acquired before the beginning of the first testing session. They were financially compensated for their time and effort. The study was approved by the Berkshire Research Ethics Committee.

#### Calculation task

Since G.M.’s task was considered impossible to solve for normal participants, even if highly proficient in mental arithmetic, we designed a simplified version that assessed accuracy and calculation speed. The task consisted of 40 multiplication problems with four different subtypes to mimic differences in the use of strategies of G.M.’s task. The four problem types were either multiplying a two-digit by a two-digit number (e.g., 34 × 76); a three-digit by a two-digit number (e.g., 669 × 86); a three-digit by a one-digit number (e.g., 539 × 7); or a four-digit by a one-digit number (e.g., 3746 × 7). The problems were controlled for mental shortcuts by excluding units of “2,” “5,” or “0,” or same units (e.g., 36 × 76). The task was presented with white letters on a black background on a computer screen. The participant had to manually enter the answer into a response window and two experimenters took the times using stopwatches on each trial. There were two different sets of problems (version A and B), one for each session. None of the problems occurred twice across versions, but the type of problems was the same. The interacting experimenter was blind to the stimulation condition and the stimulating experimenter (B.K.) noted the time when the stimulation ended, but avoided verbal communication with the participants. The task lasted beyond the stimulation duration in all cases, depending on the individual participant’s calculation speed (0–30 min). For one participant, the interacting experimenter had to leave the room due to an unusually long task duration; therefore, 12 measurements were missing. Participants were given the opportunity to rest for a minute in between trials, as some participants experienced fatigue during the task.

#### Stimulation experiment 2

Stimulation settings were as described in 2.1.4 experiment 1: procedure.

#### Procedure

Participants came to a first testing session, in which their basic mathematical abilities were assessed (WIAT®-II^UK^). As in G.M.’s case, the stimulation sessions took place on two different days (sessions 2 and 3), but with a single stimulation session (sham or active tRNS, counter-balanced across the participants) per day. After attaching the electrodes and setting the stimulation parameters, participants began the first calculation problem and had unlimited time to respond and enter each answer by hand. The duration of the task varied with the participants’ calculation speed (0–30 min). Participants received the task problem sets either in the order A/B or B/A for sessions 2/3.

### Results

**Fig. 3:**
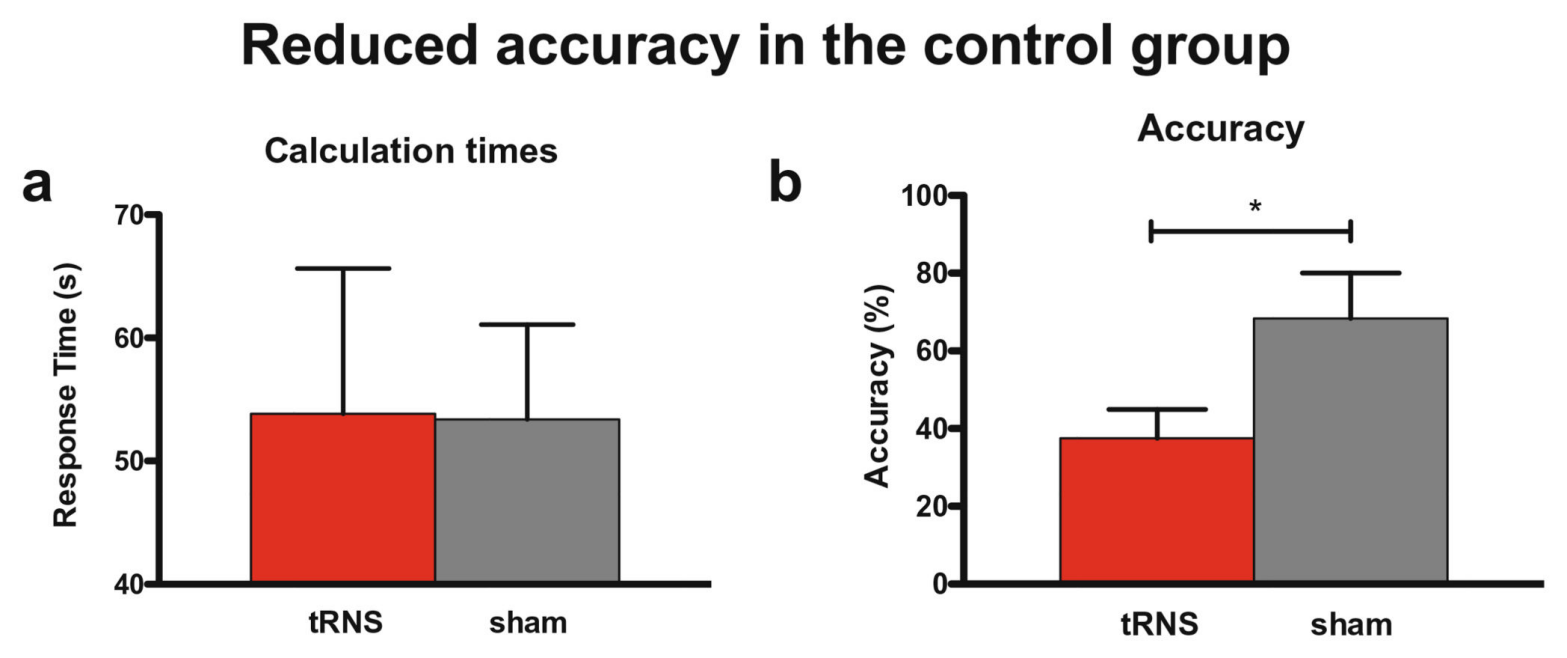
Performance of the whole participant group per condition (means and SEM). **a** Response times were not significantly different from each other, whereas **b** accuracy in terms of the percentage of correctly answered items was significantly higher in the sham compared to the real tRNS (*p* < .01**)

The number of correctly answered problems was significantly higher in the sham compared to the tRNS condition (*W* = 21; *Z* = 2.21; *p* = .03; Cohen’s *d* = 2.01) and similarly, the percentage of correct responses (accuracy; *W* = 20; *Z* = 1.99; *p* = .046; Cohen’s *d* = 1.28; Fig. 3b). Participants performed 45% better in the sham (mean accuracy = 68.33; SD = 28.75) than the tRNS condition (mean accuracy = 37.5; SD = 18.3). There was no significant difference between the number of correctly answered items or accuracy during the tRNS and immediately after the 20 min of tRNS (*W* = 12.5; *Z* = .42; *p* = .67; Cohen’s *d* = .34 and *W* = 14; *Z* = .73; *p* = .46; Cohen’s *d* = .31, respectively).

**Fig. 4:**
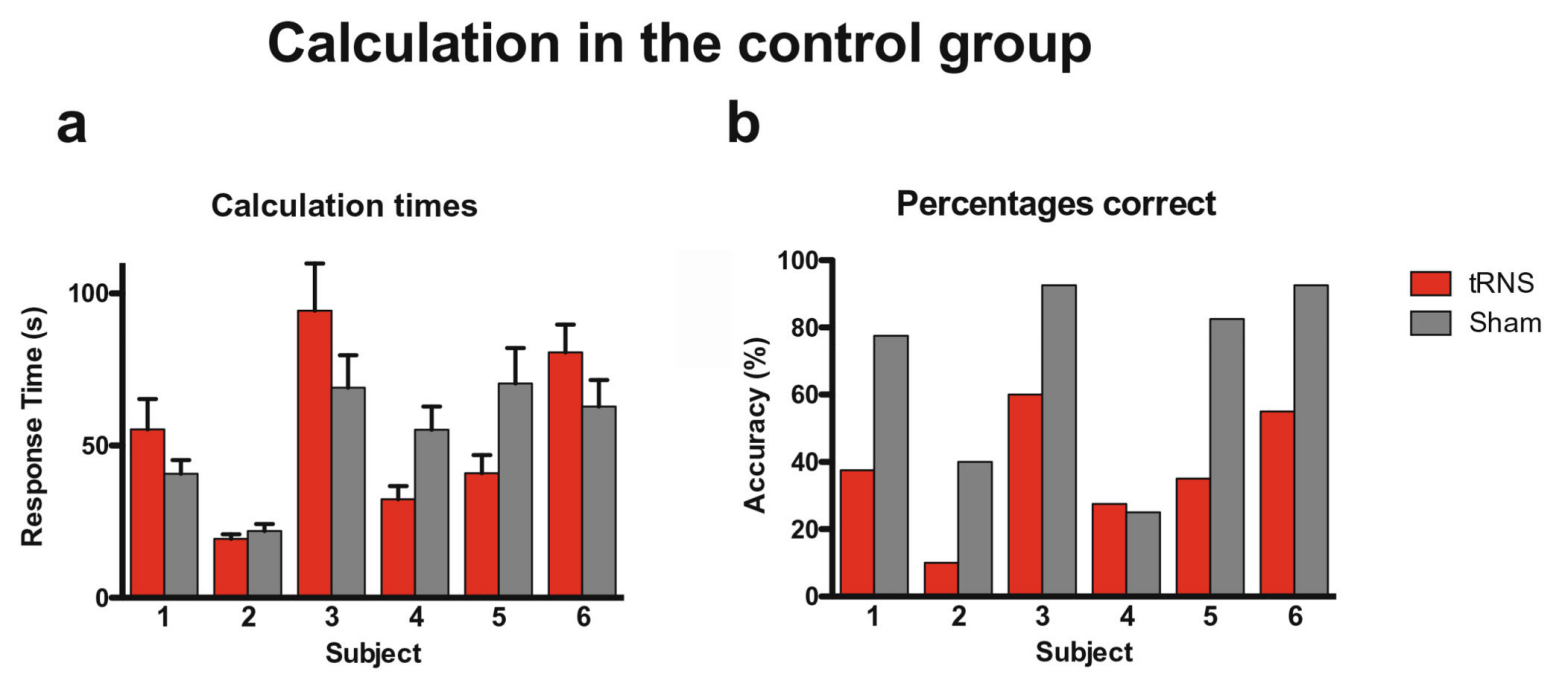
Calculation times on the correctly answered multiplication problems by participant. **a** While participant 1, 3, and 6 were faster under sham stimulation, participants 2, 4, and 5 were faster under tRNS. This effect cannot fully be explained by the counter-balanced order of calculation sheets, since participants 1, 3, and 5 had one order, and participants 2, 4, and 6 had another. **b** Accuracy; participant 4 was the only participant whose accuracy was similar under tRNS and sham, but accuracy was generally fairly low for this participant

Three hundred sixty-one average response time were available. After removal of a total of 20 outliers (10 in each condition, responses more than 2.5 standard deviations of the mean for each subject), 341 correctly answered trials were included in the analysis. We performed a nonparametric Wilcoxon related-samples ranked test on response times for correct responses. Condition (tRNS/sham) served as the within-subject factor. The conditions were not significantly different from each other (*W* = 4299.5; *Z* = −.2; *p* = .84; Cohen’s *d* = .02). Since subjects 5 and 6 demonstrated slightly lower baseline mathematical abilities (see Table 1), we additionally performed the same analysis on subjects 1–4 only. Removal of participants 5 and 6 did not change the result (*W* = 1818; *Z* = − .15; *p* = .88; Cohen’s *d* = .02; for individual performance per subject (see, Fig. 4).

### Discussion

TRNS to the bilateral DLPFC did not improve calculation performance in a calculation expert with prodigious abilities. A decrease in accuracy co-occurred with a slight improvement in calculation times, which seemed due to a speed-accuracy trade-off. The changes in calculation times and accuracy under tRNS compared to sham were, however, not significant. In contrast, in experiment 2, mathematicians showed a significant impairment in accuracy under tRNS compared to sham, but no difference in response times. While it is challenging to interpret the null results from G.M., the results from the mathematicians support the idea that the use of tRNS in individuals who are already performing at a high level can have detrimental effects. Therefore, an attempt to further improve advanced performance by using tRNS, at least with the conventional parameters used in this experiment, may compromise existing cognitive performance. As the set point theory suggests, an individual’s cognitive processing capacity may have its own homeostatic stability, and attempts to cross the limits may be counteracted by a mechanism that returns the system to its standard operating state.

To a certain degree, these findings mirror results from a transcranial direct current stimulation (tDCS) study, in which both expert and novice pianists underwent piano key stroke sequence training during a 15 min bilateral anodal-cathodal electrode configuration to primary motor cortices (Furuya et al. 2014). Pianists (all majored in piano music and had been training on the piano extensively for at least 13 years) deteriorated under the same conditions but not when anode and cathode were reversed. The authors hypothesized that the effect of tDCS here depended on the initial expertise per hand/hemisphere and that an inverted-U shape may have led to this decrease in fine motor abilities in high-performing pianists.

It should be noted that the vast majority of individuals in the population would be unable to attain the level of performance of either G.M.’s or the participants in experiment 2. In contrast, previous studies have demonstrated improvements in complex calculation in university students using tRNS (Popescu et al. 2016; Snowball et al. 2013). Both studies used five consecutive days and 20 min of tRNS to the bilateral DLPFC in a complex calculation task involving both rote learning and arithmetic computation components. TRNS was found to have beneficial effects on the speed of learning for both calculation types in 25 university students (Snowball et al. 2013). This study used two 5 × 5 cm electrodes over F3–F4 electrode positions and delivered high-frequency (100–600 Hz) of random noise to the scalp for 20 min in the active condition with 15 s ramp up and ramp down each, while the sham condition was discontinued after 30 s. The subsequent study used the same task paradigm in 32 university students (Popescu et al. 2016). In this study, 20 mins of high-frequency noise (100–640 Hz) was delivered to F3–F4 on the first 3 days of the experiment, while P3–P4 (parietal) locations were targeted for the last 2 days of the experiment. The sham condition involved the same electrode configurations but was again discontinued after 30 s. Here, tRNS improved calculation speed and learning rate and showed transfer effects to novel material for reaction times at the end of the experiment. It is therefore possible that a different range of noise frequencies (e.g., 0.1–500 Hz in this study compared to 101–640 Hz) acts on different mechanisms than higher-frequency stimulation and thereby causes oppositional effects (Saiote et al. 2013). Alternatively, it might be that the benefit of tRNS results from protocols, although the effect of impairment does not appear even during a single application in those studies (e.g., Fig. S1 in Snowball et al. 2013).

It is also important to consider that most NIBS studies are based on university students with above-average cognitive abilities. If individual tailoring of stimulation is deemed necessary, future studies need to be performed on more heterogeneous samples that also reflect the subpopulation that qualifies as the clinical target. Based on the present findings, we suggest that there might be a limit to cognitive enhancement using tRNS, at least in those with high-level mathematical abilities. Further investigations including larger sample sizes are required to draw firmer conclusions on this topic. Other cognitive domains can also give further insight into the capacity limits of the brain using NIBS.

The current study unfortunately lacked the possibility to compare G.M. to asuitable control group of normal, healthy volunteers who could perform the same task. Despite the fact that we tested a sample of mathematically highly proficient individuals, neither the arithmetic abilities nor the tasks were comparable, which, along with the sample sizes, complicates the generalizability of the effects found here. Ideally, in order to directly compare participants, G.M. and the mathematicians group would perform the same task without causing a ceiling or floor effects. For example, one study tested two participants with dyscalculia underwent numerical training under opposite parietal anodal-cathodal tDCS (Iuculano and Cohen Kadosh 2014). Since the paradigm had been tested in proficient calculators earlier (Iuculano and Cohen Kadosh 2013), it could be concluded here that one dyscalculic participant performed contrary to what was expected from the normal group when stimulated with similar parameters.

In sum, while other studies using tDCS have found that participants with the lower abilities at baseline gain most from the stimulation (Looi et al. 2016; Sarkar et al. 2014; Tseng et al. 2012), these tRNS results provide further support for the homeostatic set point hypothesis of cortical excitability, in which no further improvement, or even impairments in abilities are found upon the induction of accumulated increases in cortical excitability (Krause and Cohen Kadosh 2014; Krause et al. 2013; Siebner et al. 2004). We were therefore able to provide preliminary evidence that individuals with high expertise in mathematics show calculation impairments under tRNS, when compared to sham. These initial findings add some value to ongoing discussions about the potential consequences of neuroenhancement (Maslen et al. 2014), especially as they might be associated with cognitive costs for the individual (Iuculano and Cohen Kadosh 2013; Sarkar et al. 2014). Ideally, these results will evoke further research to examine whether the NIBS user and the application to different individuals should be considered more carefully than previously assumed.

### Conclusion

These results demonstrate that high-level cognitive abilities were not improved by tRNS, and even showed impairments, in the participants’ domain of cognitive expertise. We emphasize the importance of caution with the use of tRNS for cognitive enhancement in individuals with high-level cognitive abilities in order to prevent potential reductions or impairments in performance. Larger studies are required for more strategic research on boosting cognitive capacity in the highly skilled, but at the same time monitoring the occurrence of potential cumulative impairments in the specific population is necessary due to the potential of NIBS to alter neuroplasticity.

## Figures and Tables

**Table 1:** Participants’ mathematical abilities assessed using the Wechsler Individual Achievement Test (Wechsler 2005). The standard scores (Std) and percentiles (in brackets) are shown

Participant	Age	Numerical operations		Mathematical reasoning		Composite Mathematics	
		Std	(%)	Std	(%)	Std	(%)
1	23	123	(92)	114	(94)	121	(92)
2	30	126	(96)	126	(96)	136	(99)
3	25	121	(92)	126	(96)	131	(98)
4	35	128	(97)	123	(94)	135	(99)
5	30	122	(93)	111	(77)	118	(88)
6	25	115	(84)	102	(55)	107	(68)

Participants’ mathematical abilities assessed using the Wechsler Individual Achievement Test (Wechsler 2005). The standard scores (Std) and percentiles (in brackets) are shown
